# On Emulation of Flueric Devices in Excitable Chemical Medium

**DOI:** 10.1371/journal.pone.0168267

**Published:** 2016-12-20

**Authors:** Andrew Adamatzky

**Affiliations:** University of the West of England, Bristol, United Kingdom; Universidad Rey Juan Carlos, SPAIN

## Abstract

Flueric devices are fluidic devices without moving parts. Fluidic devices use fluid as a medium for information transfer and computation. A Belousov-Zhabotinsky (BZ) medium is a thin-layer spatially extended excitable chemical medium which exhibits travelling excitation wave-fronts. The excitation wave-fronts transfer information. Flueric devices compute via jets interaction. BZ devices compute via excitation wave-fronts interaction. In numerical model of BZ medium we show that functions of key flueric devices are implemented in the excitable chemical system: signal generator, and, xor, not and nor Boolean gates, delay elements, diodes and sensors. Flueric devices have been widely used in industry since late 1960s and are still employed in automotive and aircraft technologies. Implementation of analog of the flueric devices in the excitable chemical systems opens doors to further applications of excitation wave-based unconventional computing in soft robotics, embedded organic electronics and living technologies.

## 1 Introduction

Three designs of interaction-based computing—by using fluid streams, signals propagating along conductors and excitation wave fronts—have been conceived and evolved independently for over half-a-century.

Fluids have been used for centuries to transmit force and energy in mechanical systems. The work on using fluids for computation emerged in late 1950s early 1960s with the aim to develop reliable devices without or with minimum electronics components [1–3]. The basic principles of the fluidic devices include laminar flow of a fluid, jet interaction (where fluid flows are arranged so that small opposing jets will give various changes of direction which can be used as output signals), wall attachment (the fluid attaches to a surface within a device and continues to flow over the surface until disturbed), vortex effect and interaction with moving parts. First devices designed and fabricated in 1960s included the beam deflection, turbulence, vortex and wall attachment amplifiers, the and, not, or and xor logical elements, counters and shift registers. The fluidic devices have been used in jet sensing, programmable sequence control, flameproof equipment, machine tools control, systems operating nuclear reactor coolant, servo-control in marine applications, missile and aircraft control, artificial heart-pump, lung ventilator [1–3].

The interaction of signals traveling along one-dimensional conductors was amongst famous problems in physics, biology, and physiology for centuries, and the problemof interaction was interpreted in terms of finite state machines in 1960s. The earliest computer science related results on signal interaction can be attributed to: Atrubin [4] (a multiplier based on a one-dimensional cellular automaton), Fisher [5] (cellular automaton generator of prime numbers), Waksman [6] (firing squad synchronisation), and Banks [7] (wires and simple gates in configurations of atwo-dimensional binary-state cellular automaton). In 1982 Conway, Berlekamp and Gay demonstrated that Conway’s Game of Life cellular automaton is computationally universal [8]. The computation in the Game of Life was based on interactions between streams of gliders (compact travelling patterns of non-quiescent states) emitted by glider guns (generator of gliders). By colliding the gliders streams they implemented a functionally complete set of gates and, or and not. No fluidic devices have been mentioned in [8] yet based on the historical development of events, chances are high the computing circuits in the Conway’s Game of Life might have been inspired by jet streams interactions in early fluidic devices.

A thin-layer Belousov-Zhabotinsky (BZ) medium [9, 10] shows a rich dynamics of excitation waves including target waves, spiral waves and localised wave-fragments and their combinations. A light-sensitive BZ medium allows for optical inputs of information as parallel inputs in massive parallel processors. The medium can be also constrained geometrically in networks of conductive channels thus allowing for a directed routing of signals. A substantial number of theoretical and experimental laboratory prototypes of computing devices made of BZ medium has been reported in last thirty years. They are image processes and memory devices [11–13], logical gates implemented in geometrically constrained BZ medium [14, 15], approximation of shortest path by excitation waves [16–18], memory in BZ micro-emulsion [13], information coding with frequency of oscillations [19], onboard controllers for robots [20–22], chemical diodes [23], neuromorphic architectures [24–26, 26–29] and associative memory [30, 31], wave-based counters [32], and other information processors [33–36]. First steps have been already made towards prototyping arithmetical circuits with BZ: simulation and experimental laboratory realisation of gates [14, 15, 37–40], clocks [41] and evolving logical gates [42]. A one-bit half-adder, based on a ballistic interaction of growing patterns [43], was implemented in a geometrically-constrained light-sensitive BZ medium [44]. Models of multi-bit binary adder, decoder and comparator in BZ are proposed in [45–48]. These architectures employ crossover structures as T-shaped coincidence detectors [49] and chemical diodes [23] that heavily rely on heterogeneity of geometrically constrained space. By controlling excitability [50] in different loci of the medium we can achieve impressive results, as it is demonstrated in works related to analogs of dendritic trees [28], polymorphic logical gates [51], and experimental laboratory prototype of four-bit input, two-bit output integer square root circuits based on alternating ‘conductivity’ of junctions between channels [52].

In present paper we hybridise all three designs—jet streams interaction, glider collision, and interaction between excitation wave fronts—and uncover novel designs of the excitable medium gates inspired by fluerics. We explain Oregonator model of BZ medium in Sect. 2. We show how to generate signals in Sect. 3. Sections 4–7 present and–xor, not, nor, nor–or gates. Several implementations of diodes are discussed in Sect. 8 and delays in Sect. 9. We demonstrate analogy of a proximity sensors in Sect. 10. In Sect. 11 we show that wall attachment based flueric devices can not be implemented in the excitable chemical media. Dynamics of globally modulated wave-fragments is illustrated in Sect. 12. Section 13 outlines potential further developments in the field.

## 2 Oregonator model of an excitable medium

We use two-variable Oregonator equations [53] adapted to a light-sensitive Belousov-Zhabotinsky (BZ) reaction with applied illumination [54]. The Oregonator equations in chemistry bear the same importance as Hodgkin-Huxley and FitzHugh-Nagumo equations in neurophysiology, Brusselator in thermodynamics, Meinhardt-Gierer in biology, Lotka-Volterra in ecology, and Fisher equation in genetics. The Oregonator equations are used to model a wide range of phenomena in BZ, e.g. analysis of rotating waves [55], chaos in flow BZ [56], stochastic resonance in BZ [57], effect of macro mixing [58]. The Oregonator equations is the simplest continuous model of the BZ medium yet showing very good agreement with laboratory experiments. Let us provide few examples. A stable three-dimensional organising centre that periodically emits trigger excitation waves found experimentally is reproduced in the Oregonator model [59]. Studies of the BZ system with a global negative feedback demonstrate that the Oregonator model shows the same bifurcation scenario of bulk oscillations and wave patterns emerging when the global feedback exceed a critical value as the bifurcation scenario observed in laboratory experiments [60]. There is a good match between lab experiments on modifying excitation wave patterns in BZ using external DC field and the Oregonator model of the same phenomena [61]. The Oregonator model used in [62] to evaluate the dispersion relation for periodic wave train solutions in BZ shows agrees with experimental results. Patterns produced by the Oregonator model of a three-dimensional scrolls waves are indistinguishable from patterns produced in the laboratory experiments [63]. Excitation spiral breakup demonstrated in the Oregonator model is verified in experiments [64]. The Oregonator model can be finely tuned, e.g. adjusted for temperature dependence [65], scaled [66], modified for oxygen sensitivity [67]. Author with colleagues personally used the Oregonator model as a fast-prototyping tool and virtual testbed in designing BZ medium based computing devices which were implemented experimentally [38–42, 52].

The Oregonator equations are following

$$\begin{array}{ccc} \frac{\partial u}{\partial t} & = & \frac{1}{\epsilon }\left(u-u^{2}-\left(fv+\phi \right)\frac{u-q}{u+q}\right)+D_{u}\nabla^{2}u\\ \frac{\partial v}{\partial t} & = & u-v \end{array}$$ (1)

The variables *u* and *v* represent local concentrations of an activator, or an excitatory component of BZ system, and an inhibitor, or a refractory component. Parameter *ϵ* sets up a ratio of time scale of variables *u* and *v*, *q* is a scaling parameter depending on rates of activation/propagation and inhibition, *f* is a stoichiometric coefficient. Constant *ϕ* is a rate of inhibitor production. In a light-sensitive BZ *ϕ* represents the rate of inhibitor production proportional to intensity of illumination. The parameter *ϕ* characterises excitability of the simulated medium, this is used in tuning shape and behaviour of wave-fragments. We integrate the system using Euler method with five-node Laplace operator, zero-flux boundary conditions are specified on all sides, time step Δ*t* = 0.001 and grid point spacing Δ*x* = 0.25 (c. 0.1 mm of real space), *ϵ* = 0.02, *f* = 1.4, *q* = 0.002. The time step corresponds to c. 0.02 sec of real time and the grid space to 0.1 mm [38, 68]. The model has been verified by us in experimental laboratory studies of BZ system, and the perfect match between the model and the experiments was demonstrated in [38–41].

To generate excitation wave-fragments we perturb the medium by a square solid domains of excitation, 20 × 1 sites (unless otherwise stated) in state *u* = 1.0. Time lapse snapshots provided in the paper were recorded at every 150th time step, we display sites with *u* > 0.04; videos supplementing figures were produced by saving a frame of the simulation every 10th step of numerical integration and assembling them in the video with play rate 30 fps. All figures in this paper show time lapsed snapshots of a single wave (or two waves if there is an interaction), these are not trains of waves following each other.

**Fig 1:**
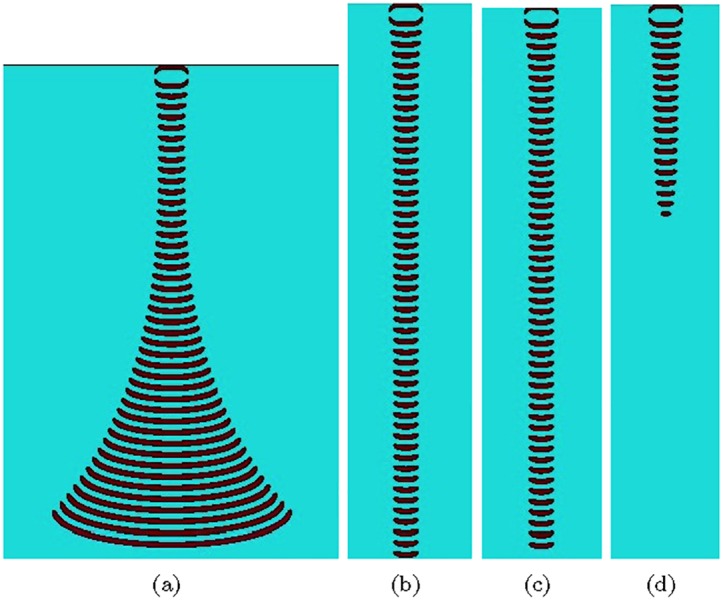
Behaviour of an initially asymmetric excitation in BZ medium for different levels of excitability. (a) Expanding wave-fragment, *ϕ* = 0.076690, (b) Shape preserving wave-fragment, *ϕ* = 0.076691, (c) Shape preserving wave-fragment, *ϕ* = 0.076698, (d) Collapsing wave-fragment, *ϕ* = 0.076699. Grid size is 302 × 449 in (a), and 108 × 495 in (bcd).

Parameter *ϕ* control excitability of the medium: if the medium is excitable, it exhibits ‘classical’ target waves when *ϕ* = 0.05. The medium is sub-excitable with propagating localizations, or wave-fragments, when *ϕ* > 0.0766. Stability of the wave-fragments can be further tuned with *ϕ*. For 0.0766 ≤ *ϕ* ≤ 0.076690 wave-fragments gradually expand (Fig 1a). The wave-fragments remain stable in long runs when 0.076691 ≤ *ϕ* ≤ 0.076698 (Fig 1b and 1c). For *ϕ* ≥ 0.076699 the wave-fragments collapse.

## 3 Generating signals

**Fig 2:**
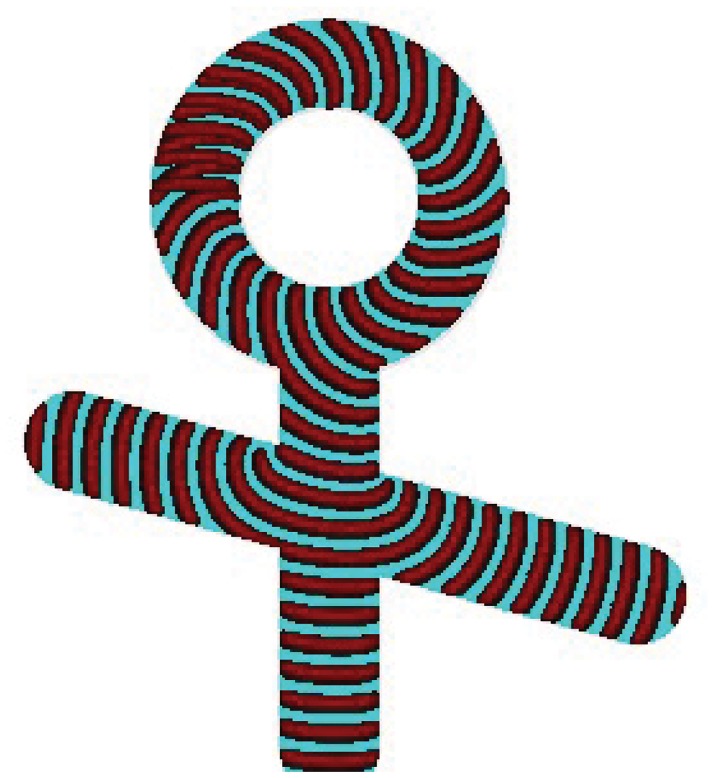
Signal generator in excitable medium. The ring and outlet are excitable channels, *ϕ* = 0.07. This is a time lapsed snapshot of a single wave-fragment, propagating along the ring and branching into two waves fragments travelling along outlets, recorded every 150*^th^* step of numerical integration. Grid size is 300 × 300 nodes. All excitable channels have width 40 nodes. Circular channel has diameter 50 nodes. Length of the vertical channel is 156 nodes, length of the slanted horizontal channel is 273 nodes.

The excitable medium’s analog of the fluidic jet stream generators is a device shown in Fig 2. It is comprised of an excitable ring [69, 70] with outlets. When the medium inside the ring is perturbed by an asymmetric excitation, e.g. a domain of 1 by 20 nodes is forced into excitable state, *u* = 0.1, *v* = 0 and a parallel domain of 1 by 20 nodes into refractory state *u* = 0, *v* = 0.1, an excitation wave-front forms and runs along the ring. If we attach outlets of excitable channels to the ring, the excitation will spread into the outlets. A frequency of the signal generation at the outlets is determined by speed of the wave front and the diameter of the ring. Speed of the wave front is determined by width of a channel (see Sect. 11). Therefore, one can achieve any frequency (in a ring with perimeter exceeding the excitation wave-length) by changing geometrical parameters of the ring.

## 4 and–xor gate

**Fig 3:**
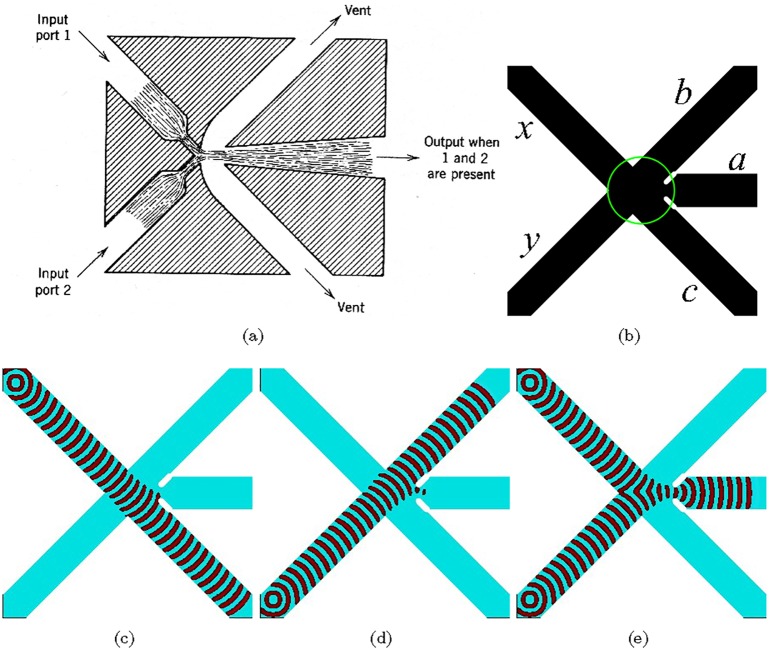
and gate. (a) Fluidic implementation [71, 72]. (b–e) Implementation in excitable medium. (b) Scheme of the gate: *x* and *y* are inputs, *a*, *b*, *c* are outputs. Channels are excitable but junction is sub-excitable: *ϕ* = 0.07 everywhere but *ϕ* = 0.0768 in the encircled domain. The excitable channels have width 40 nodes. The horizontal channels narrows to 15 nodes at the junction. Length of the horizontal channel is 114 nodes, lengths of diagonal channels are 424 nodes each.

The and gate is the most known, a par with a bistable amplifier, devices in the fluidics (Fig 3a). Two nozzles are placed at right angles to each other. When there are jet flows in both nozzle they collide and merge into a single jet entering the central outlet. If the jet flow is present only in one of the input nozzles it goes into the vent. To implement this and gate in excitable medium we cross three excitable, *ϕ* = 0.07, channels as shown in Fig 3b, and slightly illuminate the junction to make it sub-excitable, *ϕ* = 0.0768. When input *x* is excited the excitation wave propagates towards the junction and across into the output channel *c* (Fig 3c). The wave-front does not expand into the channels *a* and *b* because the junction is sub-excitable, so the wave-fragment conserves its shape. Similarly, if the input *y* is excited the excitation propagates into channel *b* (Fig 3d). When both inputs *x* and *y* are excited the wave-fragments collide with each other at the junction. They merge into a single wave-fragment which propagates into the output channel *a* (Fig 3e). The central output channel represents a conjunction of signals: *a* = *xy*. Lateral output channels represent a conjuction of one signal with negation of another signals: $b=\bar{x}y$ and $c=x\bar{y}$. By merging channels *b* and *c* into a single channel we obtain exclusive disjunction *x* ⊕ *y* thus producing a one bit half-adder. As we have already shown in [73], the gate can be further cascaded into a multiple bit full adder.

## 5 not gate

**Fig 4:**
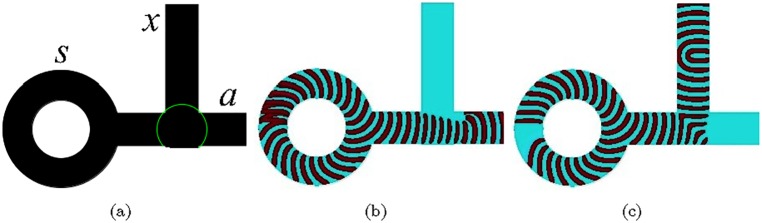
not gate implemented in excitable medium. (a) Scheme of the gate, *s* is the source of constant True, *x* is input, *a* is output. Medium is excitable apart of the junction: *ϕ* = 0.07 everywhere but area encircled in (a) is sub-excitable, *ϕ* = 0.077. All channels have width 40 nodes each. Radius of the circular channel is 50 nodes, length of the horizontal channel is 155 nodes, vertical channel 130 nodes.

Logical negation can not be implemented in passive devices, because a source of constant True is required. The signal generator (Fig 2) is used to make the not gate as demonstrated in Fig 4a. When no input signal is present the wave-front from the generator *s* exits via output *a* (Fig 4b). If excitation is generated in input *x* the wave-fragment *x* collides with the wave-fragment *s*. The fragments merge into a single wave-front (Fig 4c). This newly formed wave-front collides into the channel’s wall and annihilates. Thus $a=\bar{x}$.

## 6 nor gate

**Fig 5:**
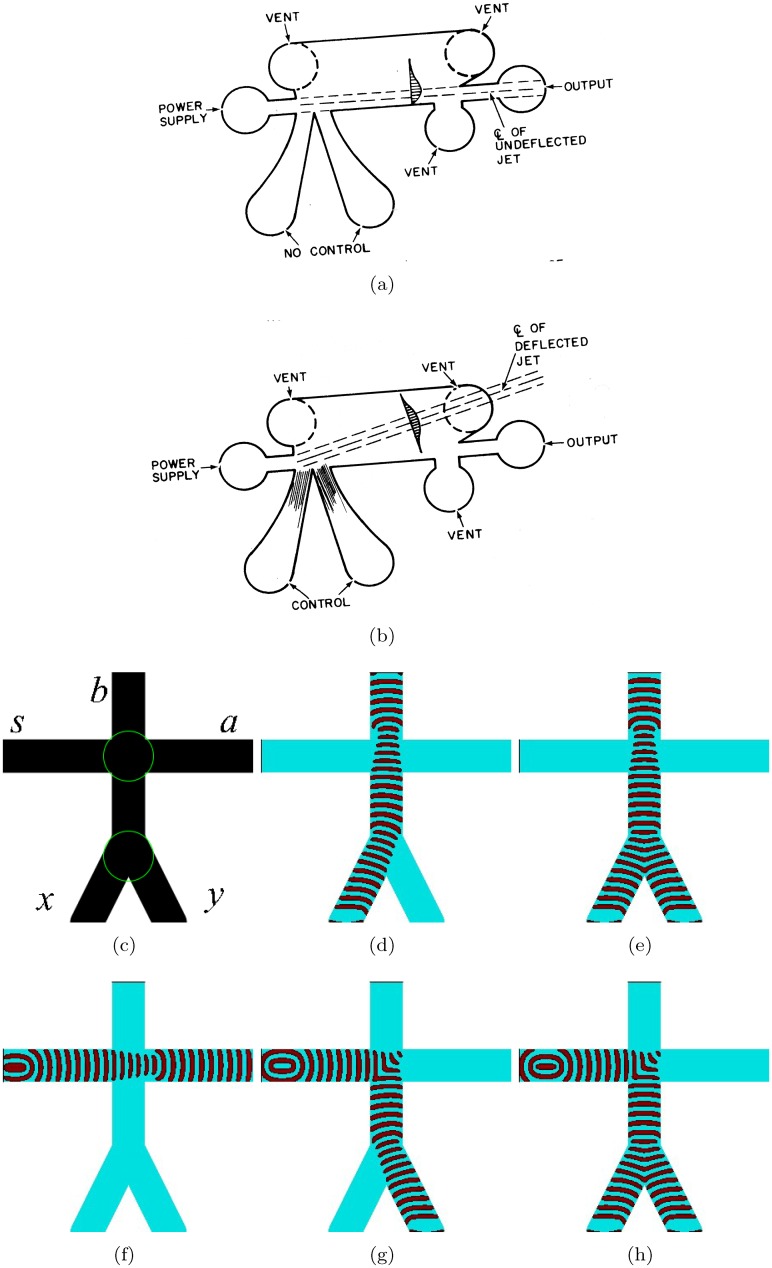
nor gate. (ab) Fluidic deflection type nor element. From [1]: (a) undeflected jet, (b) jet deflected by control stream. (c–h) Implementation in the excitable medium. (c) Scheme of the device: *s* is a power source, *x* and *y* are controls, *a* is output, *b* is auxiliary output not present in the fluidic device (ab). Channels are excitable, *ϕ* = 0.07, everywhere but junctions are sub-excitable, *ϕ* = 0.077. (d–h) Time lapsed snapshots of travelling excitations for various combinations of inputs and power source. Width of all channels is 40 nodes. The horizontal channel is 300 nodes long. Length of the vertical channel is 200 nodes. Slanted channel 111 nodes long each.

A monostable beam deflection device is comprised of a power supply, controls/inputs and vents (Fig 5a and 5b). When no inputs are present the power jet from the power source exits through the output (Fig 5a). When one or both input jets are present, the jet from the power source is deflected into the vent and discharged (Fig 5b) [1]. The power jet exits the output only if none of the input jets are present. This is nor operation. The excitable medium implementation consists of four intersecting channels (Fig 5c). The channels are excitable (*ϕ* = 0.07) and the junctions, encircled in (Fig 5c) are sub-excitable (*ϕ* = 0.077). The power source is *s* and inputs are *x* and *y*. We extend the scheme (Fig 5a and 5b) with two outputs. The output *a* in (Fig 5c) has the same purpose as the output in (Fig 5a and 5b). The output *b* produces results of additional operation. The power source can be represented by a generator described in Sect. 3; we do not show it here. When the power source is off the excitation wave-front generated at one of the inputs *x* or *y* proceeds to the output *b* (Fig 5d). When both inputs are present their wave-fragments merge and also proceed to output *b* (Fig 5e). Suppressed excitability of the medium at the junction prevents the wave-fragment from spreading to the horizontal channel. When the power source is on and no inputs are present the signal from the power source exits through the output *a* (Fig 5f). If travelling excitation is present in one (Fig 5g) or both (Fig 5h) inputs *x* and *y* the excitation wave-fragment originated at the inputs collide with the wave-fragment originated at the power source and annihilate. The output *a* represents $\bar{x+y}$ and the output *b* represents $\bar{s}\left(x+y\right)$.

## 7 nor–or gate

**Fig 6:**
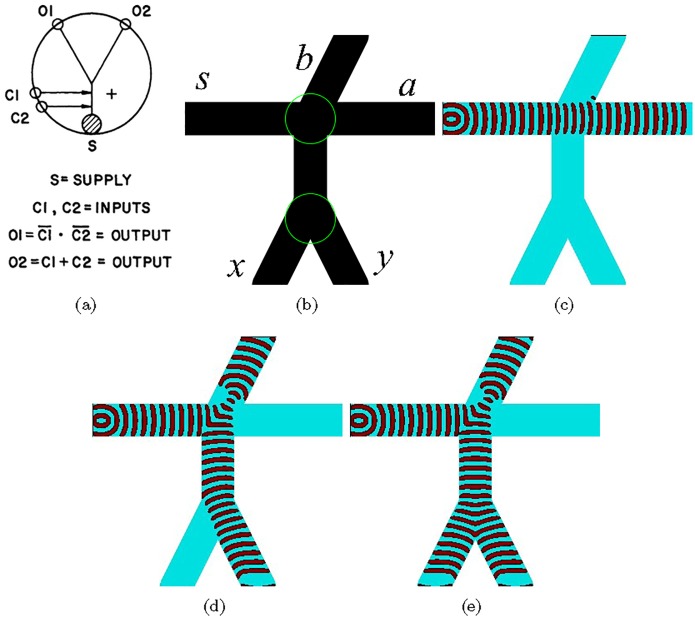
(a) A diagram of the monostable fluid nor-or amplifier. Power source is *s*, outputs are *O*1 and *O*2, controls are *C*1 and *C*2. From [74]. (b) Scheme of the device, *ϕ* = 0.07 everywhere but in encircled areas *ϕ* = 0.0763. All channels are 40 nodes wide, horizontal channel is 300 nodes long, vertical channel is 70 nodes long, all three slanted channels are 111 nodes long.

The monostable beam deflection device (Fig 5a and 5b) can be transformed into nor-or gate (Fig 6a) by adding an output outlet instead of a vent [74]. When no control jets are present the jet from the power source exits via the outlet *O*1. If one or both signal jets are present, the jet from the power source is deflected in the outlet *O*2. This device is implemented in excitable medium as follows (Fig 6b). Assume the power source is always on. When neither of the signals is present the excitation wave-front from *s* travels into output *a* (Fig 6c). If excitation wave front is generated in one of the inputs it collides with the excitation wave-front originated in the power source *s* (Fig 6c). The collided wave-fronts merge and divert into the output *b*. If excitation is generated in both inputs, the wave-fronts from *x* and *y* merge into a single wave-front before colliding with the wave-fronts *s*. The resultant wave-front collides with *s*, and is diverted into the output *b*. Thus, $a=\bar{x+y}$ and *b* = *x* + *y*.

## 8 Diodes

**Fig 7:**
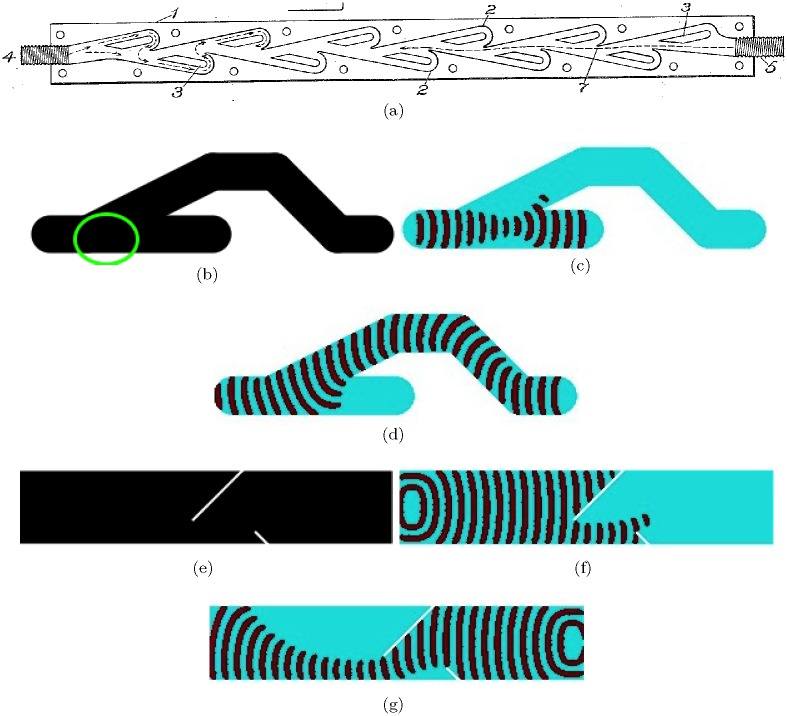
(a) Tesla diode [75], made of a casing (1), buckets (2), independent partitions (3), and input ports, or nipples (4) and (5). (bcd) Junction diode. (b) Scheme of the junction diode, all black domains have *ϕ* = 0.076 but encircled area of the junction has *ϕ* = 0.08. (c) Signal propagates in the backward flow direction; excitation wave-front travels from the left to the right. (d) Signal propagates in the forward flow direction; excitation wave-front travels from the right to the left. (efg) Parcel diode. (e) Scheme of the parcel diode, all areas of the channel are sub-excitable, *ϕ* = 0.0777, two white segments are non-excitable. (f) Signal propagates in the backward flow direction; excitation wave-front travels from the left to the right. (g) Signal propagates in the forward flow direction; excitation wave-front travels from the right to the left. Channels in (bcd) are 30 nodes wide; the configuration is bounded by 95 × 291 nodes box. Channel in (efg) is 300 nodes long and 60 nodes wide.

**Fig 8:**
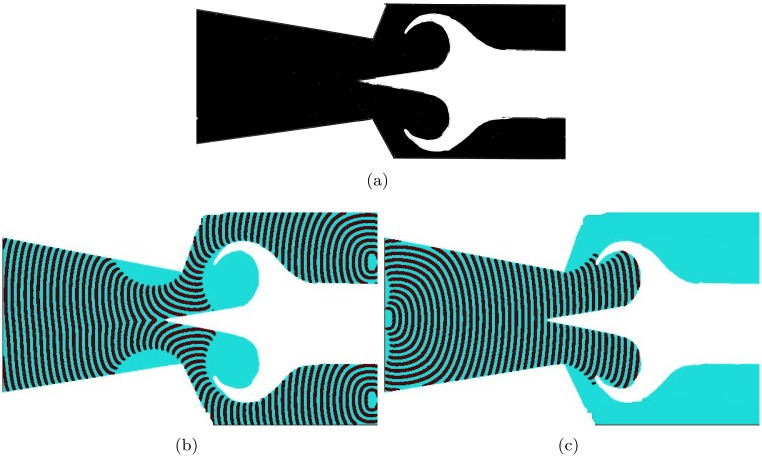
Scroll diode. (a) Scheme of the fluidic scroll diode [76] (cited by [1]). Forward flow is from the right to the left. Adapted from [1]. Black areas are excitable, *ϕ* = 0.0775, white coloured cap is non-excitable. (bd) Time lapsed snapshots of excitation wave-fragment in the scroll diode propagating in the forward direction (b) and the backward direction (d). In (b) the excitation wave-fragment travels from the right to the left. In (c) the wave-fragment travels from the left to the right. The diode configuration in (cd) is bounded by the 600 × 340 nodes box.

A fluidic diode is a two-terminal device which restricts, or even cancels, flow in one direction (backward direction). Tesla diode [75] (Fig 7a) and scroll diode [1, 76] (Fig 8a) are most known fluidic diodes (as well as vortex diode which is not discussed here).

The Tesla diode (Fig 7a), called ‘valvular conduit’ by its inventor [75], is composed of buckets and partitions arranged in such a manner that the forward flow propagates mainly along axis (4 to 5 in Fig 7a). In the backward direction (5 to 4 in Fig 7a) fluid enters the branches and loops around to oppose the main flow. Two analogies of the Tesla diode in the excitable media are the junction diode (Fig 7b, 7c and 7d) and the parcel diode (Fig 7e, 7f and 7g). The junction diode consist of a straight segment of the excitable channel ending with a cul-de-sac (analog of the vent in a fluidic device) branching to a loop-channel (Fig 7b). All channels in this diode are excitable but the junction, encircled in Fig 7b, is sub-excitable. When an excitation wave-front enters the diode in direction of the reverse flow (high resistance) the wave-front propagates across the junction without expanding into the branch and annihilates in the cul-de-sac (Fig 7c). Wave-fragment travelling in the forward direction (low resistance) propagates along the loop-channel, enters the straight segment and exits the device (Fig 7d). The parcel diode is a sub-excitable channel with two non-excitable line segments inside (Fig 7e). A wave-fragment propagating in the direction of the reverse flow collides into the parcels and partially annihilates; the small wave-fragment escaping the trap does not manage to expand sufficiently enough (due to sub-excitablity of the medium) to survive and soon collapses (Fig 7f). An excitation wave-fragment propagating in the direction of the forward flow navigates around the parcels, recovers and continues its travel along the channel (Fig 7g).

In the fluidic scroll diode the channel, or nozzle, is converging in the backward direction and enters an annular cap. In the forward direction the fluid flows through the throat and into a diffuser section. In the backward direction the fluid enters the cap and is directed back towards an incoming flow, causing a turbulence. In the excitable medium implementation of the scroll diode we assume the channel is sub-excitable and the cap is non-excitable. Excitation wave-front travelling in the forward direction splits into two wave-fragments by the cap (Fig 8b). These wave-fragments navigate around the cap, enter the diffuser and merge into a single wave-fragment which continues its travel along the channel. Excitation wave-front travelling in the backward direction collides into the cap and annihilates (Fig 8c); sub-excitability of the medium prevents the backward travelling wave-fragment from expanding into the passages around the cap.

**Fig 9:**
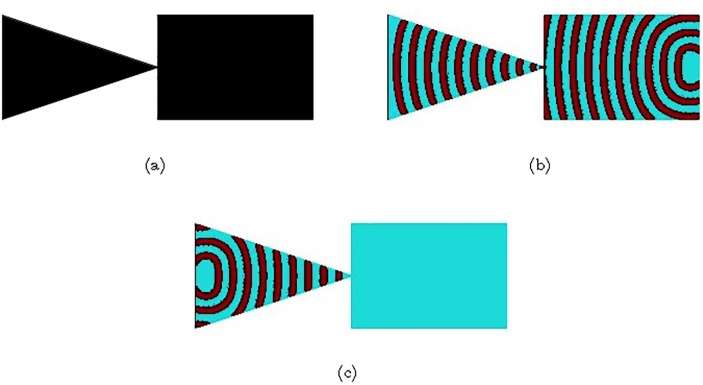
Time lapsed snapshots of a computational model of diode experimentally implemented in BZ medium in [77]. (a) Diode structure. Black areas are excitable, *ϕ* = 0.057. (b) Forward propagation. Excitation wave-front propagates from the right to the left. (c) Backward propagation. Excitation wave-front propagates from the let to the right. The rectangular channel is 150 × 100 nodes, the isosceles triangular channel has a base 100 nodes long and a median 150 nodes long.

The excitable medium implementations of the Tesla diode and the scroll diodes complement the experimental laboratory implementation of a diode in BZ medium [77]. The diode was made of two plates covered with excitable solution. The corner of the one plate was close to the plane side of another plate (Fig 9a). Excitation wave-front travelling in the forward direction reaches the contact site between the plates in a state of a planar wave, it propagates through the contact site and then continues its expanding in the triangular part of the device (Fig 9b). The wave-fragment travelling in the backward direction slows down while propagating towards the corner of the triangular plate (Fig 9c). At the contact point size of the wave-fragment becomes so small, for the level of medium’s excitability *ϕ* = 0.057, that it annihilates without crossing the contact site between the plates.

## 9 Delays

**Fig 10:**
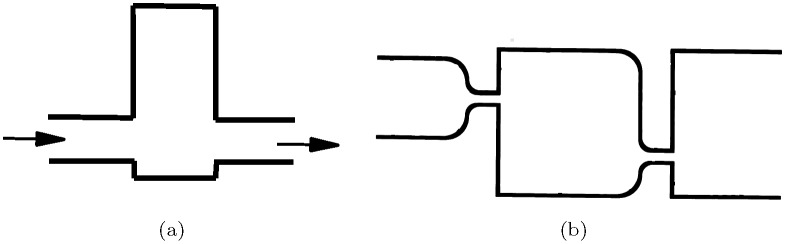
Schemes of fluidic delays. (a) Basic delay. From [78]. (b) Delay and diode. From [79].

A delay in fluidic systems is implemented as volumetric tank (Fig 10a) with input and output pipes. A step change in the input pressure on the input appears as a similar change in the output pressure on the output after a delay. The delay is caused by turbulence. The amount of the delay is determined by the volume of the tank [78].

Another version of a delay element (Fig 10b) combines orifices and volume to have a low impedance in one direction of flow (from the left to the right in Fig 10b) and a high impedance in the opposite direction of flow (from the right to the left) [79]. The impedance provides a phase shift during transient flow. The phase shift contributes to the retarding of the fluid flow, thus introducing a time delay of the flow. The device can also act as a diode and a pressure divider.

**Fig 11:**
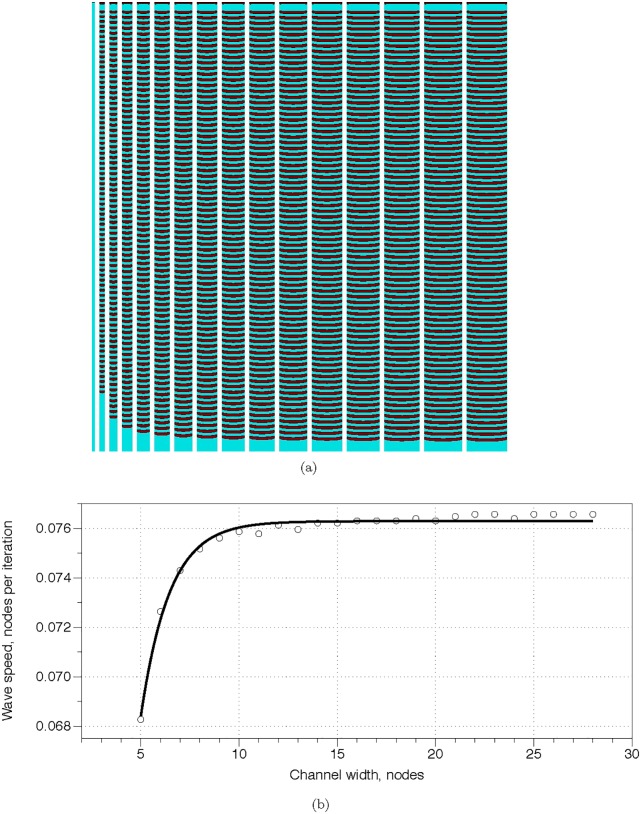
Excitation waves are delayed in narrow channels. (a) Time lapsed snapshots of excitation waves propagating in 24 excitable channels width from 5 to 29 nodes, length 900, *ϕ* = 0.07, *ϕ* = 0.024; the waves are initiated at the top ends of the channels. (b) Speed of wave propagation versus width of a channel. Data from computational experiments are shown by circles, solid line is the exponential approximation *v* = 0.0762964 − 0.2431048 * *e*^−0.6853214*w^.

**Fig 12:**
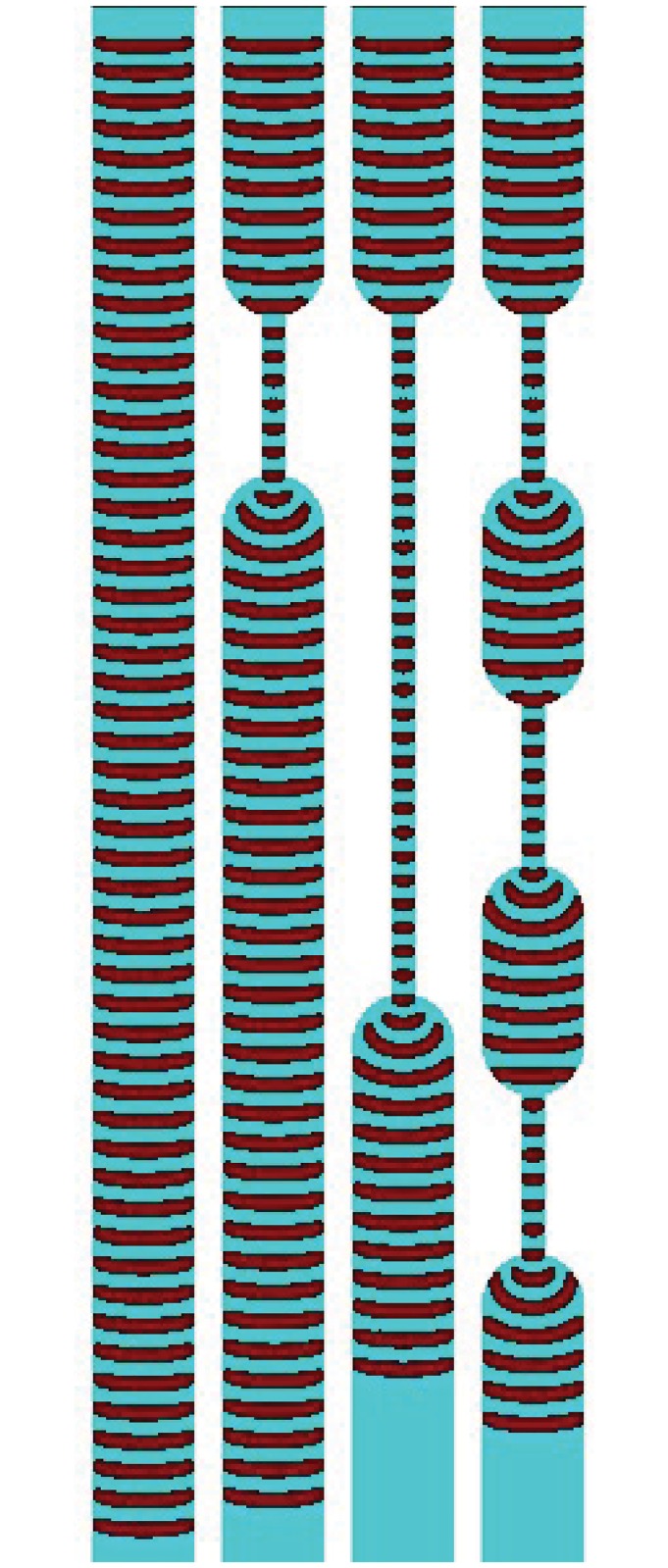
Examples of delays, *ϕ* = 0.07, *ϕ* = 0.024. Waves are initiated at the top ends of the channels. Width of regular channel is 40 nodes, of the delay-channels 10 nodes, total length of each channel is 600 nodes.

There is no exact analog of pressure in excitable medium. We can assume the ‘pressure’ is always constant. Wave-fronts in excitable channels are compact finite objects positions of which can be detected at any given moment of time. Thus delay in excitable medium is a late, compared to a channel without the delay element, arrival of wave-fragment at given site of a channel. A speed of excitation wave in a channel is proportional to a width of the channel [15, 80, 81]. We illustrate this in computer experiments. Fig 11a shows time lapsed snapshots of waves propagating in 24 channels with width varying from 5 to 29. In channels with less than 6 nodes width no excitation sustains: this is well in agreement with experimental laboratory findings [80]. The channels are excitable: *ϕ* = 0.07. We calculate a speed of a wave as a function of channel’s width (Fig 11b). Let *w* be a width of an excitable channel, *v* be a speed of wave propagation, then *v* = 0.0762964 − 0.2431048 * *e*^−0.6853214*w^, with coefficient of determination *R*^2^ = 0.9896. Excitation wave does not propagate in a channel narrower than 6 nodes, and effect of width becomes relatively negligible when channel width exceed 11 nodes. Examples of simple conductors of excitation incorporating delays are shown in (Fig 12): on halt of the simulation waves in the channels travelled 592, 580, 530 and 551 nodes.

## 10 Sensor

Interrupted jet sensor, see e.g. [82], is a device comprised a single nozzle positioned in spaced registry with an inlet. The nozzle is supplied with pressurised fluid, the fluid is ejected form the nozzle as a free jet towards the inlet. If there is an object between the nozzle and the inlet, the jet steam becomes disturbed and a phase shift in pressure occurs, which is reflected by the inlet [83, 84]. Applications of the interrupted jet sensors are limited to counting and fabric positing devices, because it is impractical to place large objects between the jet and the inlet. The implementation of an analog of the interrupted jet sensor such as sensor in an excitable medium would be a trivial task.

**Fig 13:**
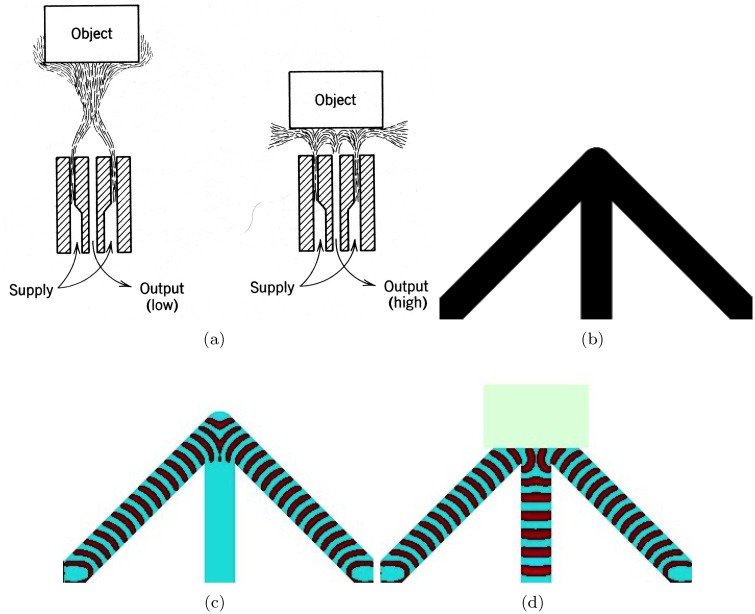
Proximity sensor in fluidics and excitable medium. (a) Converging jet sensor. From [72]. (bc) Sensor implemented in excitable medium *ϕ* = 0.07. (b) No object sensed. (c) Sensed object is represented by high-illumination domain. All channels have width 30 nodes. The vertical channel is 160 nodes long, the slanted channels are 221 nodes long.

Converging jet sensor [85] detects a proximity to an objects via the flow pressure differences (Fig 13a). The sensor has an annular orifice for ejecting a annular jet of fluid. The is also an inlet to connect output volume to the interior volume of the device. When the output jet is disturbed by a proximity of an object the output pressure increases sharply and operates the response device [85]. The analogous to the converging jet sensor device is implemented in the *modulated* (see Sect. 15) excitable media by crossing two excitable channels and placing a vertical ‘sensing’ channel between them (Fig 13b). The starting *ϕ* = 0.07 but it its continuously updated during simulation to keep total level of excitation at a level fixed when wave-fragments started to propagate in the input channels. An excitation wave-front in the ‘sensing’ channel indicates presence of an object. A sensed object is represented by a domain of high-intensity illumination, which inhibits the excitation. If no object present the wave-fronts initiated at the lateral channels reach the intersection side, collide and annihilate (Fig 13c). If tip of the sensor is in proximity of a sensed object—rectangular domain in Fig 13d—the wave-fronts are partially reflected by the object, merge together into a single wave-front and propagate into the middle (sensing) channel.

## 11 Bistable devices

**Fig 14:**
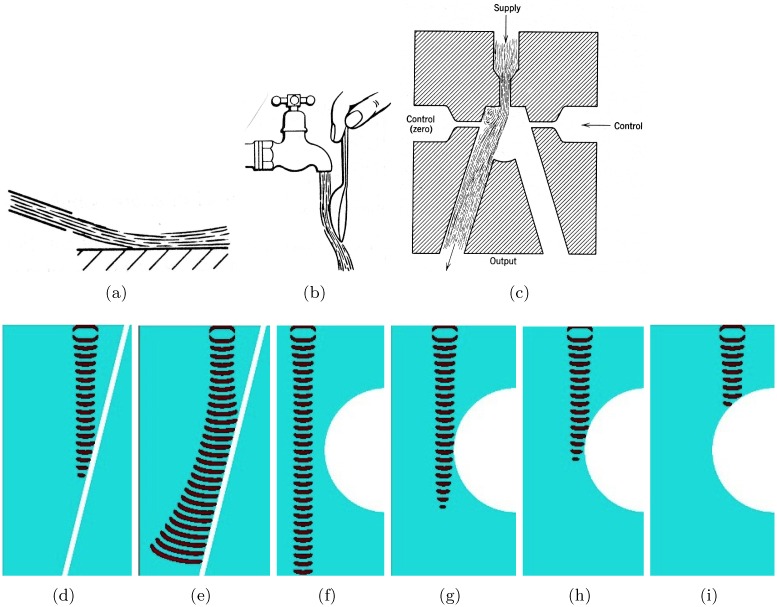
(a) The fluid jet is attached to a surface. From [72]. (b) The fluid jet remains attached when the surface curves away. From [72]. (c) Bistable wall attachment amplifier. From [72]. (d) Time lapsed snapshots of the localised wave-fragment in sub-excitable medium colliding to a flat surface, *ϕ* = 0.076691. The white line segments are non-excitable. (e) Time lapsed snapshots of the expanding wave-fragment in nearly excitable medium colliding to a flat surface, *ϕ* = 0.076690. The white line segments are non-excitable. (f–i) Wave-fragment interacts with a non-excitable convex domain. (f) Wave-fragment propagates at some distance form the domain without touching it. (gh) Part of the wave-fragment touches the convex domain. (i) The wave-fragment completely collides into the domain.

A jet flow attached itself to a nearby surface (Fig 14a) and remains attached even when the surface curves away from the initial direction of the power jet (Fig 14b). This is the Coanda effect [86]. The wall attachment of the jet happens due to a difference in space from the jet to an object’s surface. The effect is used to implement bistable amplifiers (they are called amplifiers because stronger power jets are deflected by weaker control jets) and flip-flop elements in fluidic devices. The exemplar bi-stable device has a power jet source, two output channels and two control channels (Fig 14c). The power jet entering the junction, or a branching site, with no controls present would become attached to a wall of one the channels, chosen arbitrarily. The jet attracts air in the space between itself and one wall, and makes a vacuum in the space between itself and another wall [2]. By activating a control jet one can divert the power source jet to another channel, where the jet got attached and continues to be attached after the control signal is switched off. This allows us to implement flip-flop devices in fluidic circuits which are key components of fluidic computers, e.g. register shift devices [87, 88].

We did not find a way to implement bistable devices in a sub-excitable medium. A localised excitation wave-front in sub-excitable medium does not attach itself to a surface (Fig 14d) but annihilates. This is because a level of the medium excitability is not high enough to allow the wave-fragment to restore its size. In a medium with increased excitability, where wave-fragments expand, an apparent ‘attachment’ might take place (Fig 14e) however this is caused by expansion of the wave-front not because of the physical interaction of the wave-fragment with the surface. The localised wave-fragment keeps it is shape intact when travels in a medium without obstacles (Fig 14f). If such a wave-fragment collides, even partially, with an obstacle the fragment did not restore its shape but annihilates (Fig 14g, 14h and 14i).

## 12 Dynamic modulation of excitability

**Fig 15:**
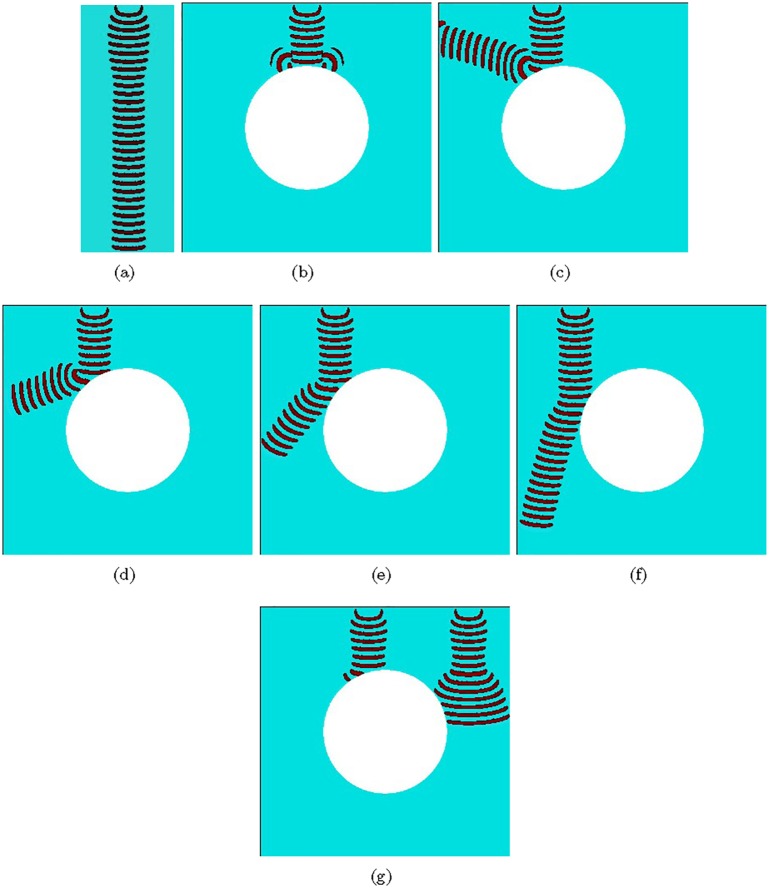
Modulated wave-fragments. (a) Wave-fragment propagating south in a uniform medium. (b–f) Collision a wave-fragment with illuminated disc. (e) Dynamics of two wave-fragments. Grid size is 300 × 300 nodes, disc of radius 150 nodes is centred. Wave-fragments are initiated by exciting a domain of 2 × 20 nodes near north edge of the grid.

A modulation is a change of the medium’s excitability on the fly to prevent the excitation wave-fragment from collapsing or exploding. The modulation is implemented as follows. When the wave-fragment just forms, we calculate activity level *γ*, as a number of nodes with *u* > 0.1. On further steps of simulation we calculate activity level *α* and compare it with the standard activity level *γ*. If *α* > *γ* we decrease excitability of the medium by increasing *ϕ* as *ϕ* → *ϕ* + 0.001; if If *α* < *γ* we decrease *ϕ* by 0.001. The similar method of modulation was used in the experimental laboratory routing of excitation wave-fragments in BZ medium [89]. Example of the modulated wave-fragment is shown in Fig 15a, slight oscillation in the wave-fragment size is visible. When a non-modulated shape preserving compact wave-front collides to or brushes by the non-excitable domain the wave-fragment collapses (Fig 14g, 14h and 14i). The modulated wave-fragment annihilates only in a head-on collision with a non-excitable domain (Fig 15b). In scenarios of a partial contact with the non-excitable domain the wave-fragment recovers and reflects pf the domain (Fig 15c–15f). The deficiency of the global modulation is that when two or more wave-fragments present they might implicitly compete for the ‘quote of activity’ allocated: large fragments would become larger and small fragments would collapse, as illustrated in Fig 15g.

## 13 Discussion

The jet streams in flueric (fluidic without moving parts) devices and excitation wave-fronts in excitable media have different physical nature. Despite this we demonstrated that it is possible to emulate most common flueric devices in the excitable media, the Belousov-Zhabotinsky (BZ) system: power sources (emulated by excitable rings with outlets), delay elements, diodes, not, and and nor gates, and proximity sensors. Two basic principles of fluidic devices have been emulated with the excitation wave-fragments: laminar flow and jet interaction. We have been unable to implement bistable devices because the excitation wave-fragments do not attach the walls as the jet streams do. There may be other ways, not inspired by Coanda effect, to make the bistable devices in the sub-excitable media. Further studies can focus on analogous implementations of wall-attachment based devices, laminar turbulent effect, vortex effect and vortex diodes, and moving part devices where excitation wave-fragments can manipulate objects. The application domain for excitable media computing and sensing devices is presently very limited, comparing to the applications of fluidic devices (which have already over half-a-century track record of industrial implementations) however the field of unconventional computing and novel materials is rapidly changing and more potential applications and laboratory prototypes emerge.

Most experimental prototypes of collision-based computing devices, inspired by Fredkin-Toffoli conservative logic [90], Margolus soft-balls model [91], Squier-Steglitz particle machine [92] and its advancement to solitonic computing [93], rely on precise timing of the collision between signals. Examples include interaction of wave-fragments in light-sensitive Belousov-Zhabotinsky media [94], swarms of soldier crabs [95], growing lamellipodia of slime mould *Physarum polycephalum* [43, 96], crystallisation patterns in ‘hot ice’ [97], peristaltic waves in protoplasmic tubes [98], and jet streams in fluidic devices [99], competing patterns propagation in channels of communication with a Life-like CA [100]. When Boolean values are represented by localised, finite size, patterns—the accuracy of synchronisation depends on the size of the patterns. For example, if two wave-fragments in BZ medium collide not ‘perfectly’ but with an offset more than a half-wave length the output of the gate will be ineligible. To make these designs asynchronous we must find an analog of a latch, this could be a scope of further studies.

Future focal points of prototyping computing, sensing and actuating devices using interacting excitation wave-fronts are soft robotics with gels impregnated with excitable chemical systems [101–104], self-propulsive BZ droplets and chemical robots [105, 106], BZ medium encapsulated in arrays of micro-droplets [107, 108], controlling micro-fluidic systems with excitable media [109], nano-scale realisations of reaction-diffusion computers [110], and implementation of computing circuits with wave-fronts of electrical potential travelling in bioengineered living tissue [111].

## Supporting Information

S1 File Video supporting Fig 2. Signal generator in action.
(MOV)

S2 File Videos supporting Fig 3. (A) and gate in cation, for *x* = 0 and *y* = 1 and (B) *x* = 1 and *y* = 1.
(ZIP)

S3 File Videos supporting Fig 4. (A) not gate in action for *x* = 0 and (B) *x* = 1.
(ZIP)

S4 File Videos supporting Fig 6. nor-or gate in action for (A) *x* = 0 and *y* = 0 (B) *x* = 1 and *y* = 0 (C) *x* = 1 and *y* = 1.
(ZIP)

S5 File Videos supporting Fig 7. (A) The junction diode forward propagation. (B) The junction diode backward propagation. (C) The parcel diode forward propagation. (D) The parcel diode backward propagation.
(ZIP)

S6 File Videos supporting Fig 8. (A) Forward wave-front propagation in the scroll diode and (B) backward propagation.
(ZIP)

S7 File Video supporting Fig 12. Delays in action.
(MOV)

S8 File Videos supporting Fig 13. (A) Sensor without any object in proximity. (B) The sensor with an object in proximity.
(ZIP)
